# The Effects of QEEG-Informed Neurofeedback in ADHD: An Open-Label Pilot Study

**DOI:** 10.1007/s10484-012-9191-4

**Published:** 2012-03-24

**Authors:** Martijn Arns, Wilhelmus Drinkenburg, J. Leon Kenemans

**Affiliations:** 1 Research Institute Brainclinics, Bijleveldsingel 34, 6524AD Nijmegen, The Netherlands; 2 Department of Experimental Psychology, Utrecht University, Utrecht, The Netherlands; 3 Janssen Research and Development, Pharmaceutical Companies of J&J, Beerse, Belgium

**Keywords:** QEEG, Neurofeedback, ADHD

## Abstract

In ADHD several EEG biomarkers have been described before, with relevance to treatment outcome to stimulant medication. This pilot-study aimed at personalizing neurofeedback treatment to these specific sub-groups to investigate if such an approach leads to improved clinical outcomes. Furthermore, pre- and post-treatment EEG and ERP changes were investigated in a sub-group to study the neurophysiological effects of neurofeedback. Twenty-one patients with ADHD were treated with QEEG-informed neurofeedback and post-treatment effects on inattention (ATT), hyperactivity/impulsivity (HI) and comorbid depressive symptoms were investigated. There was a significant improvement for both ATT, HI and comorbid depressive complaints after QEEG-informed neurofeedback. The effect size for ATT was 1.78 and for HI was 1.22. Furthermore, anterior individual alpha peak frequency (iAPF) demonstrated a strong relation to improvement on comorbid depressive complaints. Pre- and post-treatment effects for the SMR neurofeedback sub-group exhibited increased N200 and P300 amplitudes and decreased SMR EEG power post-treatment. This pilot study is the first study demonstrating that it is possible to select neurofeedback protocols based on individual EEG biomarkers and suggests this results in improved treatment outcome specifically for ATT, however these results should be replicated in further controlled studies. A slow anterior iAPF at baseline predicts poor treatment response on comorbid depressive complaints in line with studies in depression. The effects of SMR neurofeedback resulted in specific ERP and EEG changes.

## Introduction

The development of personalized medicine in psychiatry has received increased interest, with a quest for biomarkers that can be used to predict treatment outcome to specific therapies. Stratification of patient subgroups is one of the basic approaches to personalized medicine. This can be achieved for example by measures of brain function such as the EEG. In ADHD it has been reported that ADHD patients with excess frontal theta EEG power (Arns et al. 2008; Clarke et al. 2002; Satterfield et al. 1971) and excess frontal alpha EEG power (Arns et al. 2008; Chabot et al. 1999) are more likely to respond to stimulant medication. Furthermore, a low-voltage EEG occurs more often in ADHD as compared to controls Arns et al. (2008). Conceptually, stratification in these 3 ‘sub-groups’ has been interpreted as sub-groups of ADHD patients exhibiting a lower and more instable vigilance regulation, while the ADHD symptoms are explained by so-called ‘vigilance auto-stabilization behavior’ (Hegerl et al. 2010; Sander et al. 2010). This, in turn would be consistent with the efficacy of stimulant medication in these sub-groups.

Another reported neurophysiological sub-group is composed of patients showing an excess beta or beta spindling (Arns et al. 2008; Chabot and Serfontein 1996; Clarke et al. 2001) who were reported to respond to stimulant medication by Clarke et al. (2003) whereas Arns et al. (2008) reported a lack of a significant improvement on impulsivity and inattention (ATT) after stimulant medication. Finally, ADHD patients with a slowed individual Alpha Peak Frequency (iAPF) do not respond to stimulant medication (Arns et al. 2008) which presumably characterizes a non-specific trait of non-response to various treatments because deviations in this measure have also been found in non-responders (NR) to antidepressants (Ulrich et al. 1984) and repetitive transcranial magnetic stimulation (rTMS) in depression (Arns et al. 2010, in press; Conca et al. 2000).

Recently a meta-analysis on the effects of neurofeedback in the treatment of ADHD has been published in which it was concluded that neurofeedback resulted in large and clinically relevant effect sizes (ES) for ATT and impulsivity and a low to medium ES for hyperactivity (Arns et al. 2009). Furthermore, several studies have demonstrated that the effects of neurofeedback are maintained over 6 months follow-up (Gevensleben et al. 2010; Leins et al. 2007; Strehl et al. 2006). However, several recent studies employing placebo controlled designs failed to find a difference between neurofeedback and sham-neurofeedback consisting of a non-contingent feedback control condition (Lansbergen et al. 2011; Perreau-Linck et al. 2010). Although both comprised small sample sizes (Perreau-Linck et al. (2010): N = 4 and Lansbergen et al. (2011): N = 8) and had methodological limitations such as the use of auto-tresholding and unconventional QEEG based protocols (Lansbergen et al. 2011) these studies warrant more research into the specificity of neurofeedback in ADHD.

In a pioneering study by Monastra et al. (2002) only ADHD patients with a deviating theta/beta ratio were selected and treated with theta/beta neurofeedback, which resulted in a substantial ES of 1.8 on ATT, which for that reason was excluded from the meta-analysis by Arns et al. (2009). Therefore, in this study we aimed to personalize the neurofeedback protocol based on the individual EEG pattern—as described above—to investigate if such an approach leads to better clinical results as compared to Arns et al. (2009). Additionally we expect that patients with a slow iAPF will be NR to neurofeedback. Furthermore, pre- and post-treatment EEG and ERP changes will be investigated to investigate if neurofeedback results in any neurophysiological changes suggestive of a neurophysiological normalization, which is assumed to be the rationale behind neurofeedback.

## Methods

### Participants

This study is an open-label pilot study. All files from patients seen in our clinic (Brainclinics, Nijmegen, The Netherlands) between August 12th 2008 and September 12th 2010 were screened. Patients were screened for ADHD or ADD by a clinical psychologist using a structured interview (MINI Plus Dutch version 5.0.0, for adults or MINI KID for children) during intake. During intake, every 10th session and outtake a DSM-IV based self-report scale for ADHD symptoms (Kooij et al. 2005) was assessed. Mood disorders are very common in (adult) ADHD (38 %: Kessler et al. 2006) hence the Becks depression Inventory (BDI) was also assessed when comorbid depressive complaints were present at screening. Only subjects with a primary diagnosis of ADHD/ADD were included in the study. Only results at pre-treatment, mid-treatment and at post-treatment will be reported. All patients signed an informed consent form before treatment was initiated.

### Pre- and Post-assessments: QEEG and ERP’s

EEG and ERP recordings were performed using a standardized methodology and platform (Brain Resource Ltd., Australia), details of this procedure have been published elsewhere (Arns et al. 2008; Williams et al. 2005) and details of reliability, validity and across site-consistency of this EEG and ERP procedure have been published here (Clark et al. 2006; Paul et al. 2007; Williams et al. 2005). This methodology has been used in more than 250 publications and an overview of these methods and publications can be found on www.brainnet.net.

In summary, patients were seated in a sound and light attenuated room, controlled at an ambient temperature of 22 °C. EEG data were acquired from 26 channels: Fp1, Fp2, F7, F3, Fz, F4, F8, FC3, FCz, FC4, T3, C3, Cz, C4, T4, CP3, CPz, CP4, T5, P3, Pz, P4, T6, O1, Oz and O2 (Quikcap; NuAmps; 10–20 electrode international system). Data were referenced to averaged mastoids with a ground at Fpz. Horizontal eye movements were recorded with electrodes placed 1.5 cm lateral to the outer canthus of each eye. Vertical eye movements were recorded with electrodes placed 3 mm above the middle of the left eyebrow and 1.5 cm below the middle of the left bottom eyelid. Skin resistance was <5 K Ohms for all electrodes. A continuous acquisition system was employed and EEG data were EOG corrected offline. The sampling rate of all channels was 500 Hz. A low pass filter with attenuation of 40 dB per decade above 100 Hz was employed prior to digitization. The auditory event-related potential was measured during an auditory oddball task. During EEG recording patients were exposed to a series of high and low pitched tones. They were asked to press a button with their left and right index finger in response to the high-pitched tone, while keeping their eyes fixed on a red dot presented on a computer screen in front of them. Subjects were asked to sit quietly.

### QEEG Informed Neurofeedback Protocols

The QEEG was used to establish the neurofeedback protocol by visual inspection of the raw EEG followed by inspection of the deviating Z-scores after comparison to the Brain Resource International Brain database. More details on this procedure for the use in ADHD have been published by Williams et al. (2010). The QEEG informed selection of neurofeedback protocols in line with the four ADHD subtypes presented in the introduction (Frontal Theta, Frontal Alpha, Low Voltage and Excess Beta) was based on the decision rules as outlined below. These subtypes and recommendations are in line with the EEG Phenotype approach (see: Johnstone et al. (2005) for more details and background). For most clients two neurofeedback protocols were used throughout the treatment, with the goal to use at least one of the well-established protocols (SMR/Theta or Theta/Beta) and one additional protocol based on other QEEG findings and symptoms. The locations for C3 and C4 for the SMR protocol were established using TMS to localize the area where a visual response of the musculus abductor pollicis (thumb movement) was observed, in order to also personalize the neurofeedback location to be exactly localized above the sensori-motor strip.

The following decision rules were used to obtain QEEG-informed neurofeedback protocols:

1. *Frontocentral Theta/(beta) protocol*: If excess fronto-central theta was observed then the midline site (Fz, FCz or Cz) where this activity was maximal was chosen and the exact theta frequency band was determined from the QEEG report by inspecting the Z-scores for single hertz bins in the theta frequency range. In these patients hence a theta/beta protocol was used with an additional reward on beta (15–20 Hz). When there was beta-excess, only theta would be downtrained and no beta reward was used. When theta was normal but beta was decreased only beta was rewarded.
2. *Frontocentral alpha protocol*: If there was excess fronto-central alpha (especially during eyes open) then the midline site where this activity was maximal was chosen and next this activity was downtrained. If there was no excess beta activity or beta spindles then a beta reward was also used.
3. *Beta*-*downtraining protocol*: If excess beta or beta spindles were present then the site where this activity was maximal (Z-score) was identified and selected as training site. The exact training frequency was established from the QEEG single Hz bin Z-scores and this frequency was specifically downtrained. No further inhibits or rewards were used.
4. *A low*-*voltage EEG*: If this type of EEG was observed, then an ‘SMR protocol’ was used (either rewarding SMR spindles with a 0.25 s. duration, or SMR/theta at C3/C4). When there was also a lack of alpha power during eyes closed, alpha uptraining during Eyes Closed at Pz (*Alpha*-*uptraining protocol*) was added, as suggested by Johnstone et al. (2005).
5. If there were no clear QEEG deviations and/or if sleep problems were a main complaint, then an ‘SMR protocol’ was used (the side was chosen based on the location where the 12–15 Hz activity was lowest).

In all protocols EMG inhibits were employed whereby the EMG (55–100 Hz) had to be kept below 5–10 μV. An overview of all protocols used in this study is depicted in Table 1.

**Table 1:** This table shows the neurofeedback protocols received by the different patients

ID	Frontocentral T/(b)	Frontocentral Alpha/(b)	Beta downtraining	SMR Protocol	Alpha uptraining
1		Pz (EO)		1	
2			1	1	1
3				1	1
4			1	1	
5		1		1	
6				1	1
7	1			1	
8			1	1	
9	1		1		
10			1		
11				1	1
12		1			
13	1			1	
14		1		1	
15	1			1	
16	1		1		
17	1			1	
18	1			1	
19			1		
20	1				1
21				1	1

Note that ID 1 received alpha downtraining at Pz (EO) since alpha was most specifically increased at that site

All other patients received standard versions of the protocols as outlined above

### Neurofeedback Treatment

Treatment was carried out by a masters level psychologist specialized in neurofeedback, supervised by the first author. Sessions took place 2–3 times a week, for 20–30 min provided in 5 min blocks separated by a 2 min pause. The wireless Brainquiry PET 4.0 (Brainquiry B.V.) and BioExplorer software (CyberEvolution, Inc.) were used to provide visual feedback (bargraphs or neuropuzzles) and auditory feedback. Tresholds were set to achieve a 75–80 % reward per training contingency. For discrete SMR training the threshold was aimed at providing 1-min reward during a 5-min period, or adjusted consequently.

### Data Analysis

#### Clinical Outcome

All patients treated have been included in the analysis, including patients who dropped out or who did not respond to treatment.

ADHD patients were classified into the following groups based on outtake data:

- *Responder (R)*: At least a 50 % reduction on one or both subscales of the ADHD self-report rating scale [ATT or Hyperactivity/Impulsivity (HI)] at outtake.
- *Drop*-*out (DO)*: When a patient did not take more than 20 sessions and could not be classified a responder. A last observation carried forward (LOCF) procedure was used to handle these data in that the last available scores (at session 10) were used as ‘outtake’.
- *Non*-*responder (NR)*: A patient not meeting criteria for being a ‘responder’ who finished more than 20 sessions of neurofeedback.

### EEG and ERP Variables

The employed method used for calculation of the iAPF has been published before (Doppelmayr et al. 1998; Lansbergen et al. 2011; Arns et al. in press) but in summary consisted of EOG correction of eyes open (EO) and eyes closed (EC) EEG data (Gratton et al. 1983); filtering (1–40 Hz), segmentation in 8 s. epochs and manual de-artifacting using Brain Vision Analyzer 2.0 (BVA). The FFT power spectrum (6–13 Hz for children and 7–13 Hz for adults) from EO was deducted from the FFT power spectrum from and the maximum (representative of maximum alpha suppression) was established at P3, Pz, P4, O1, Oz or O2. Furthermore, the average iAPF at anterior sites (F3, Fz and F4) was scored at the frequency with maximum alpha suppression. Data from the SMR (12–15 Hz), Alpha (8–12 Hz) and Beta band (15–20 Hz) were extracted using an FFT for pre- and post-treatment EEG’s for EO and EC.

Conventional ERP averages were calculated at Pz. The peaks (amplitude and latency) of the N100, P200, N200 and P300 for the target waveforms of the ERP component were identified (relative to a pre-stimulus baseline average of −300 to 0 ms).

### Statistical Analysis

A repeated measures ANOVA with factor time (3 levels, pre-; mid-; and post-treatment) and between factor Child–Adult was used to investigate the effects on ATT and HI. One-way ANOVA’s were used to investigate whether there were any baseline differences between R and NR on ATT, HI, BDI scores and iAPF and posterior and anterior iAPF were correlated with ATT, HI and BDI.

Pre- and post-treatment differences on ERP components were assessed using a repeated measures ANOVA with factor time (pre- and post-treatment) and for EEG power (alpha, SMR and Beta) using a repeated measures ANOVA with factor time (pre- and post-treatment) and a factor site (9 channels: FC3, FCz, FC4, C3, Cz, C4, CP3, CPz and CP4) and the within subject factor condition (EO or EC).

The within group ES for the neurofeedback effects were calculated using MetaWin 2.1 and these were plotted against the effect sizes from the meta-analysis obtained for the whole meta-analysis (Arns et al. 2009) and the ES for Monastra et al. (2002).

## Results

### Clinical Outcome

Table 2 shows the sample characteristics and the neurofeedback protocols used. Note that 1/3th of the sample consisted of children and 2/3th consisted of adults with ADHD/ADD, and approximately half of the sample was diagnosed with ADD (N = 11) and the other half with ADHD (N = 10). Six patients were medicated with methylphenidate, one with dextro-amphetamine, one with citalopram and one with risperidon.

**Table 2:** Sample characteristics and neurofeedback protocols used in the present study

Sample characteristics	
Age	29,95 (SD: 16,19) years
Gender	8 female/13 male
Children/adults	7 children/14 adults
Medicated	9/21
ADD/ADHD	11/10
Number of sessions	33.62 (SD: 16.09)
Neurofeedback protocols	
SMR protocol	15/21
Theta/(beta) protocol	6/21
Beta-downtraining protocol	7/21
Frontal alpha protocol	3/21
Alpha-uptraining protocol	6/21

**Fig. 1:**
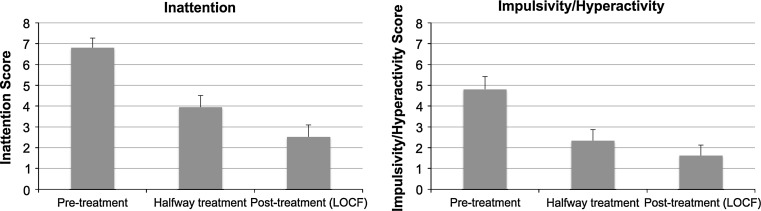
Clinical effects over time for the total group of ADHD/ADD patients at pre-treatment, halfway treatment and post-treatment (averages plus SEM) for ATT and HI. All time effects were significant (*p* ≤ .001)

**Fig. 2:**
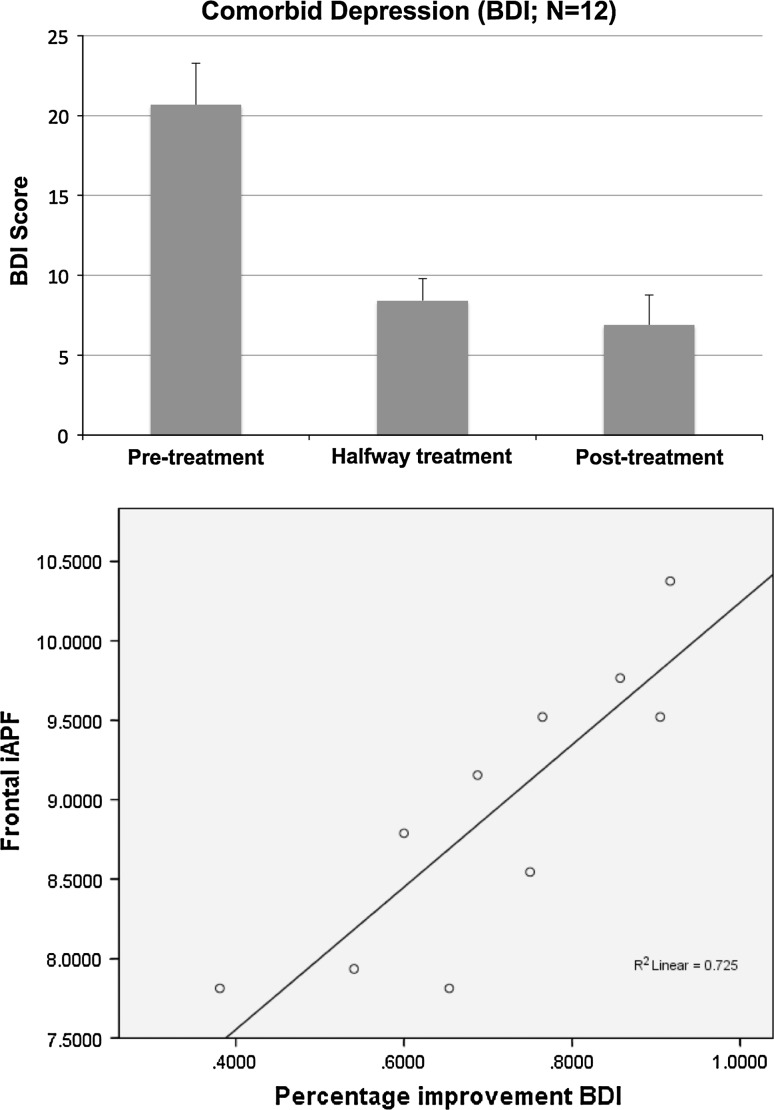
Improvement on comorbid depressive symptoms for the patients across time (time effects: *p* = .003; *Left*) and the significant correlation between the frontal iAPF and the percentage improvement in BDI scores (*p* = .002; r = 0.851; *Right*)

General response rate was 76 % (16/21), with three patients classified as a NR (14 %) and 2 as a DO (10 %). See Figs. 1, 2 for an overview of the results. Figure 1 demonstrates the effects on ATT and HI, whereas Fig. 2 shows the effects on the BDI reflective of comorbid depressive symptoms. For Fig. 2 only data from 12 subjects were available, since they initially presented with elevated depression scores whereas the remaining nine subjects did not.

The analysis only revealed significant effects of time (ATT: *p* = .000; F = 16.377; DF = 2, 18; HI: *p* = .001; F = 10.795; DF = 2, 18; BDI: *p* = .003; F = 14.517; DF = 2, 7) but no significant ATT X Child–Adult or impulsivity X Child–Adult interactions, suggesting the effects of neurofeedback were similar for children and adults. Also see Fig. 1 for the scores on ATT and HI over time. There were no differences between R and NR on ATT, HI and BDI at baseline.

**Fig. 3:**
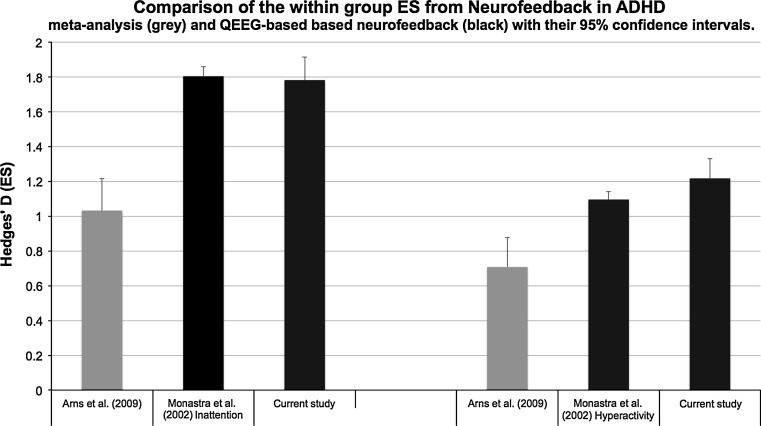
ES for the different studies mentioned in the introduction and the ES obtained from the current study, with on the left ES for ATT and on the right ES for hyperactivity. Note that ES for hyperactivity for this study was based on a combined HI scale

Figure 3 below shows that the within subject ES from the current study for ATT was 1.78 and for HI was 1.22, compared to the within subject ES obtained from the meta-analysis (Arns et al. 2009) and the Monastra et al. (2002) study.

### iAPF

Two subjects exhibited a low-voltage EEG that did not allow calculation of a reliable iAPF. Therefore, BDI data were available for 12 patients (who at baseline demonstrated an increased BDI score) and for 10 patients a correlation with the iAPF could be established. Furthermore, 2 ADD subjects had a score of 0 on HI hence no percentage change scores for HI was available for these two subjects.

No correlations were found between the iAPF (both anterior and posterior) with percentage improvement on ADHD ATT and HI. Furthermore, one-way ANOVA demonstrated no differences between R and NR on posterior and anterior iAPF, lending no support to the finding that NR displayed lower iAPF’s.

A significant correlation was found between the anterior iAPF and the percentage improvement on the BDI (*p* = .002; r = 0.851, DF = 10) suggesting that patients with a slow iAPF improved much less on comorbid depressive complaints. Note that there were no correlations between the improvements on the BDI and ATT or HI hence this could not explain the clinical improvements. Figure 2 depicts the improvement over time on the BDI scores and also the correlation between baseline anterior iAPF and improvement on the BDI after neurofeedback.

### Pre- and Post-treatment Effects of Neurofeedback on QEEG and ERP’s

Due to the open-label nature of this study, pre-treatment and post-treatment data for EEG and ERP’s were only available for six R treated with SMR neurofeedback.

**Fig. 4:**
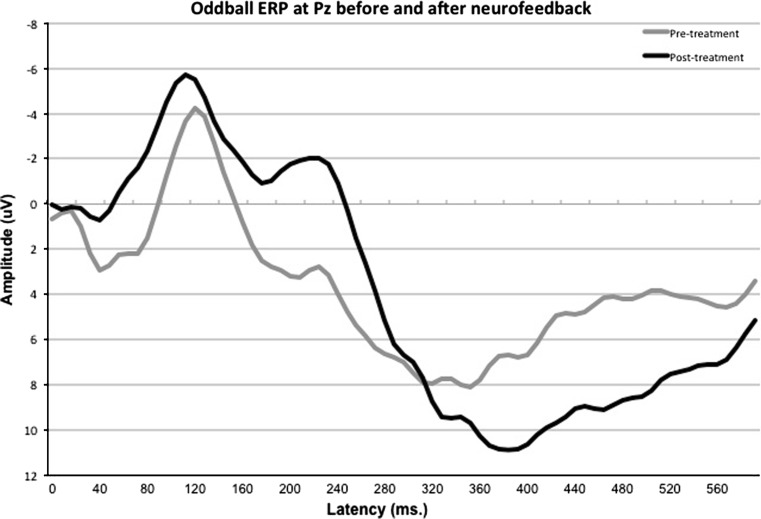
Oddball ERP at Pz before and after treatment for a sub-group of patients who have all been treated with SMR neurofeedback. Note the clear increased N200 and P300 amplitudes after treatment

There were no time effects neither for N100 and P200 amplitudes and latencies, nor for the N200 and P300 latency (all *p* > .18). There was a significant time effect for N200 amplitude (*p* = .014; F = 13.861; DF = 1, 5) and P300 amplitude (*p* = .004; F = 24.190; DF = 1, 5). In Fig. 4, the oddball ERP at Pz is visualized, demonstrating that there was a clear increase in N200 and P300 amplitude after neurofeedback treatment.

**Fig. 5:**
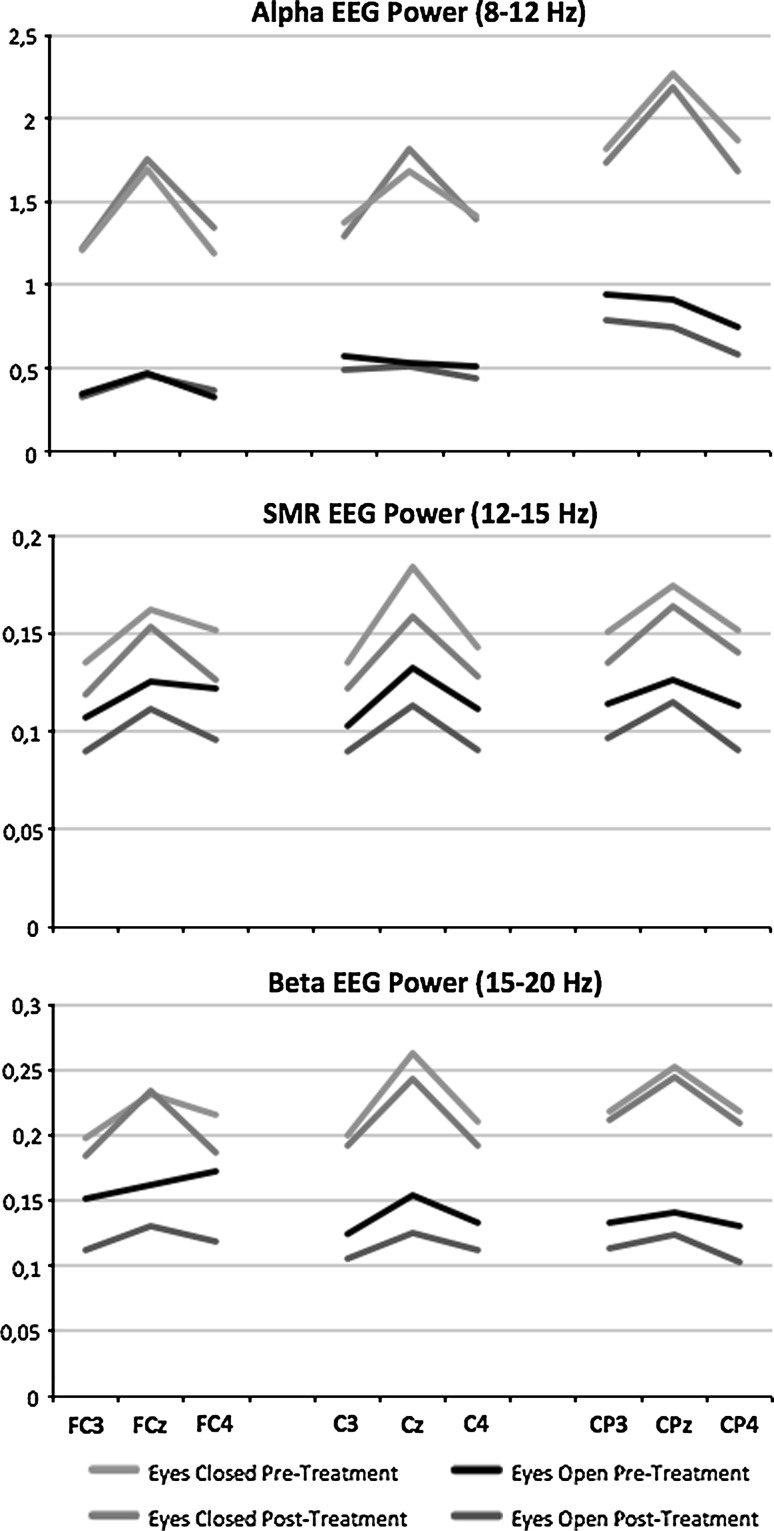
Pre- to post-treatment changes in EEG power for SMR power—which was trained using neurofeedback—and the neighboring frequency bands alpha and beta. Note the specific decrease in SMR power from pre- to post-treatment for both eyes open and eyes closed EEG, which is specific for only the SMR frequency band

The repeated measures ANOVA for SMR power demonstrated a significant effect of time (*p* = .009; F = 10.254; DF = 1, 10) and site (*p* = .033; F = 12.010; DF = 8, 3). No Time × Condition, Site × Condition, Time × Site or Time × Site × Condition interactions and no main effect of condition were found. For alpha power and beta power there were neither significant main effects nor significant interactions. In Fig. 5 these data are depicted and as can be seen SMR power was significantly *decreased* post treatment. This figure further demonstrates the specificity of the effect for the SMR band only and not in the neighboring frequency bands alpha and beta.

## Discussion

This pilot-study is the first study to investigate in a systematic way the effects of QEEG-informed neurofeedback in ADHD. It was found that neurofeedback resulted in significant improvements on ATT, HI and comorbid depressive complaints and the response rate was 76 %. The ES obtained in this study were identical to the ES reported by Monastra et al. (2002) for ATT and were almost double the ES reported in the meta-analysis (Arns et al. 2009). In comparison, a recently conducted meta-analysis on the effects of stimulant medication in ADHD found an ES of 0.84 for Ritalin on ATT (Faraone and Buitelaar 2009). Therefore, these results suggest that personalizing the treatment to the individual QEEG improves clinical outcomes, most clearly for ATT. Regarding the effects on HI it is difficult to draw conclusions. Arns et al. (2009) already pointed out that the effects of neurofeedback on hyperactivity are to a large part due to non-specific treatment effects. In this study we only had a combined measure of HI making a direct comparison difficult and possibly explaining the slightly larger ES as compared to the other studies (see Fig. 3). Obviously, these results require replication in order to confirm these findings.

We did not find a clear relationship between a slow iAPF and treatment outcome on ADHD relevant measures as hypothesized. However, we did find that a slow anterior iAPF at baseline was associated with a smaller decrease of comorbid depressive complaints as measured on the BDI in agreement with the depression literature [tricyclic antidepressants: Ulrich et al. (1984), rTMS: Arns et al. (2010, in press); Conca et al. (2000)] supporting the notion that a slow anterior iAPF at baseline is related to worse treatment outcome on depressive complaints. In this study only few patients had an iAPF of 8 Hz or lower, whereas in Arns et al. (2008, 2010) this group was larger. Hence, in this sample the representation of slow iAPF’s might have been too low to find a clear relationship between a slow iAPF and treatment outcome on ADHD rating scales. Therefore, the conclusion that neurofeedback can be considered an effective treatment for those patients with a slow iAPF and who do not respond to stimulant medication is unjustified at this moment. More research with larger samples is required to further investigate this issue.

### Pre- to Post-treatment Effects

In a sub-group of R who all underwent an SMR protocol we were able to demonstrate specific pre- to post-treatment improvements such as increased N200 and P300 amplitude and specific effects only related to the SMR EEG frequency band. The N200 has been related to stimulus discrimination (Näätänen and Picton 1986) and the P300 to attention and memory updating (for review see: Kenemans and Kähkönen 2011) and both have been found to be reduced in ADHD (for review see: Barry et al. 2003). Therefore, the finding of increased N200 and P300 amplitude suggests a normalization in underlying neural circuitry related to stimulus discrimination and attention/memory updating. Normalization of ERP components in ADHD as a result of neurofeedback has been reported by several other authors as well (Heinrich et al. 2004; Kropotov et al. 2005; Wangler et al. 2011), therefore this finding provides further support of the specificity of SMR neurofeedback in this sub-group of patients.

Regarding post-treatment EEG changes, patients exhibited *decreased* SMR power post-treatment whereas the neurofeedback aimed at *increasing* this frequency band. The observed effects in the EEG were specific to the narrow SMR frequency band of 12–15 Hz and were not found in the neighboring alpha and beta frequency bands, which suggests the effects are specific to the frequency band trained (see Fig. 5).

Similar findings were observed in an earlier study by Pineda et al. (2008). They observed that children with autism demonstrated impaired mu-suppression when observing movement. In their double-blind neurofeedback study they rewarded mu rhythm (10–13 Hz) and found that mu-suppression was significantly *improved* after treatment. So by uptraining this frequency they found that children were better able to suppress that frequency. This finding hints at the notion that SMR neurofeedback serves as a procedure to teach people voluntary control over specific EEG frequencies, rather then structurally upregulate this EEG activity. This would be more in line with the Slow Cortical Potential neurofeedback (SCP) approach where children with ADHD learn to self-regulate their SCP towards both positivity and negativity (Heinrich et al. 2004; Strehl et al. 2006). In an earlier BCI study in which we compared SCP and SMR as a means of achieving voluntary control, we also demonstrated that healthy volunteers are able to self regulate SMR in a comparable way as subjects can self-regulate their SCP’s. In this study subjects had to randomly enhance or suppress their SMR relative to baseline, and 30 % gained control by SMR suppression whereas 40 % gained by control by SMR enhancement (Kleinnijenhuis et al. 2008) demonstrating that subjects develop individual strategies to achieve control.

### Limitations

This pilot-study lacked a (double-blind) control group hence it cannot be ruled out that the effects were due to non-specific treatment effects as pointed out in previous studies (Lansbergen et al. 2011; Perreau-Linck et al. 2010). Furthermore, in contrast to most other studies, in this study neurofeedback was carried out as ‘treatment as usual’ and often patients had to pay out-of-pocket to cover the costs of neurofeedback. This might have potentially led to the higher ES as well. The comorbid depressive symptoms were only assessed in patients with initial deviating scores on the BDI and pre- and post-EEG and ERP’s were only collected in a sub-group of responders, which limits the generalizability of the findings in this study. Therefore, future controlled studies should assess scales such as the BDI in all subjects and conduct pre- and post-treatment EEG and ERPs in all subjects to replicate and confirm the findings from this study.

Finally, calculating an ES based on pilot study data is not as reliable as calculating these on large RCT’s (Kraemer et al. 2006), hence caution should be taken in interpreting the ES reported in this study. The reported ES in Fig. 3 only provides a rough indication of the effects and an RCT is required to further substantiate this ES for QEEG informed neurofeedback.

### Summary

This pilot-study provides support for the possibility to personalize neurofeedback treatment to the individual QEEG using a limited set of decision rules whereby most patients are still treated with one of the well investigated neurofeedback protocols (SMR/Theta or Theta/Beta neurofeedback), resulting in high response rates and a relatively high ES on ATT. Furthermore, specific neurophysiological improvements (increased N200 and P300 ERP amplitudes and decreased SMR) were obtained in a sub-group of patients who were treated with SMR neurofeedback. Future studies employing randomized double-blind placebo controlled designs and larger sample sizes are required to replicate these findings. The decision rules employed in this study could be easily used for designing a study employing more objective means of QEEG-based protocol selection.
